# A protective effect of inflammatory bowel disease on the severity of sclerosing cholangitis

**DOI:** 10.3389/fimmu.2024.1307297

**Published:** 2024-03-06

**Authors:** Friederike Stumme, Niklas Steffens, Babett Steglich, Franziska Mathies, Mikolaj Nawrocki, Morsal Sabihi, Shiwa Soukou-Wargalla, Emilia Göke, Jan Kempski, Thorben Fründt, Sören Weidemann, Christoph Schramm, Nicola Gagliani, Samuel Huber, Tanja Bedke

**Affiliations:** ^1^Section of Molecular Immunology and Gastroenterology, I. Department of Medicine, University Medical Center Hamburg-Eppendorf, Hamburg, Germany; ^2^Hamburg Center for Translational Immunology (HCTI), University Medical Center Hamburg-Eppendorf, Hamburg, Germany; ^3^Department of General Visceral and Thoracic Surgery, University Medical Center Hamburg-Eppendorf, Hamburg, Germany; ^4^Center of Diagnostics, Institute of Pathology, University Medical Center Hamburg-Eppendorf, Hamburg, Germany; ^5^Martin Zeitz Center for Rare Diseases, University Medical Center Hamburg-Eppendorf, Hamburg, Germany

**Keywords:** primary sclerosing cholangitis, inflammatory bowel disease, Mdr2 knock out, microbiota, colitis

## Abstract

**Background:**

Primary sclerosing cholangitis (PSC) is a chronic liver disease marked by inflammation of the bile ducts and results in the development of strictures and fibrosis. A robust clinical correlation exists between PSC and inflammatory bowel disease (IBD). At present, published data are controversial, and it is yet unclear whether IBD drives or attenuates PSC.

**Methods:**

*Mdr2*-deficient mice or DDC-fed mice were used as experimental models for sclerosing cholangitis. Additionally, colitis was induced in mice with experimental sclerosing cholangitis, either through infection with *Citrobacter rodentium* or by feeding with DSS. Lastly, fibrosis levels were determined through FibroScan analysis in people with PSC and PSC-IBD.

**Results:**

Using two distinct experimental models of colitis and two models of sclerosing cholangitis, we found that colitis does not aggravate liver pathology, but rather reduces liver inflammation and liver fibrosis. Likewise, people with PSC-IBD have decreased liver fibrosis compared to those with PSC alone.

**Conclusions:**

We found evidence that intestinal inflammation attenuates liver pathology. This study serves as a basis for further research on the pathogenesis of PSC and PSC-IBD, as well as the molecular mechanism responsible for the protective effect of IBD on PSC development. This study could lead to the discovery of novel therapeutic targets for PSC.

## 1 Introduction

Primary sclerosing cholangitis (PSC) is a chronic liver disease marked by inflammation of the bile ducts and results in the development of strictures and fibrosis (1). Progressive damage to the liver and bile ducts results in a median liver transplant-free survival period of 12-20 years after initial diagnosis (2). Of note, PSC is highly linked with inflammatory bowel disease (IBD), in which 60-80% of patients also display clinically evident IBD (2–5). Furthermore, we have recently shown that a high proportion of people with PSC, even without clinical evident IBD, show molecular signs of intestinal inflammation characterized by immune cell infiltration and expression of proinflammatory cytokines in intestinal biopsies (6). Interestingly, PSC-associated IBD presents differently from Crohn’s disease (CD) and Ulcerative colitis (UC), with predominant right-sided colitis or pancolitis, backwash ileitis, rectal sparing, and overall milder symptoms (7). Furthermore, individuals with PSC-associated IBD have distinct microbiota compositions from those with IBD only (8–11). Overall, these studies indicate a link between PSC and IBD, and suggest a potential involvement of the gut-liver axis.

Indeed, previous studies suggest an important role for the gut-liver axis in several diseases going beyond PSC (12, 13). It has been demonstrated that barrier defects during colitis can lead to the migration of gut microbes from the colon to the liver (12). Hence, the suggestion was made that the liver might function as an effective vascular barrier, eliminating commensal microorganisms that have breached the intestinal vascular circuits (12). In line with this, we found that microbes and T cells migrate to the liver during intestinal inflammation (13). Moreover, it has been demonstrated that bacterial translocation can trigger immune cell activation within the liver (14). Accordingly, studies in mice suggest that the migration of activated T cells from the intestine to the liver through common chemokines might drive PSC (15, 16). In line with these studies, it has been demonstrated that colitis promotes cholangitis in a mouse model where the transfer of ovalbumin (OVA)-specific OT-1 CD8^+^ T cells induces inflammation in the liver (17). Thus, these studies support the hypothesis that IBD drives PSC. However, there is no clear correlation between the severity of PSC and IBD (7). Moreover, and in contrast to the hypothesis that IBD drives PSC, it has recently been shown in an experimental mouse model of sclerosing cholangitis, that colitis rather improves hepatocyte injury and liver cholestasis, but does not, per se, drive PSC. Furthermore, the presence of intestinal inflammation in PSC patients has been linked to extended transplantation-free survival, suggesting a possible protective influence against the progression of the disease (18).

Taken together, these studies show a rather pathogenic effect of IBD on the development of PSC. However, at present, published data are controversial, and it is still unclear whether IBD drives or attenuates PSC. Recently, Gui et al. provided data indicating that IBD may also have a protective effect on PSC severity. Specifically, they discovered that intestinal inflammation ameliorates cholestatic liver damage. This outcome occurred regardless of changes in microbial bile acid metabolism induced by colitis. Instead, it was facilitated through hepatocellular NF-κB activation by lipopolysaccharide (LPS), which suppresses bile acid metabolism both in experimental setups and living organisms *in vitro* and *in vivo* (18). However, these data were based on the use of a single mouse model for IBD and sclerosing cholangitis. Furthermore, they assessed one cohort of PSC-IBD patients. In our study, we aimed to test, if this observed effect is mouse model specific. We specifically aimed to use a mouse model of IBD that is not dependent on a chemical induction (i.e. DSS), and which is known to additionally affect the liver (19, 20). Finally, we compared the cholangitis severity in our cohort (6) of individuals with PSC-IBD.

## 2 Material and methods

### 2.1 Patients

PSC was diagnosed using MRCP or ERCP or liver biopsy based on traditional criteria. In accordance with standard clinical practice, all patients underwent routine assessment for IBD-related symptoms. To determine the diagnosis of IBD in these patients, the evaluating physician conducted a comprehensive assessment that encompassed their clinical symptoms (quantified by CDAI or Mayo score), the assessment of routine histology (qualitative assessment via hematoxylin/eosin staining and the endoscopy results). Individuals with elevated IBD scores were described as PSC-IBD patients, and individuals with IBD in remission were excluded. All patients provided informed written consent. The study was carried out with the approval of the ethical commission of the medical association Hamburg (PV4444, PV7106). Liver stiffness measurements were acquired using FibroScan (Echo-Sense, Paris, France), as previously reported (21). Spleen length was measured via ultrasound (22). IBD disease severity was assessed through CDAI (for Crohn’s Disease patients) or Mayo Score (for all other IBD patients) and merged into a simplified IBD score ranging from 0 to 4 as described in (23).

### 2.2 Mice

*Mdr2^-/-^* mice and C57BL/6 wild type mice were bred and housed under specific pathogen-free conditions (SPF) at the animal facility of the University Hospital Hamburg-Eppendorf. C57BL/6 germ-free *wild type* mice were bred and housed at the University Hospital Hamburg-Eppendorf under germ-free conditions. Age and sex-matched littermates were used for all experiments. Food and water were provided *ad libitum*. Animal experiments were approved by the local committee (N35/2013, N17/2012, N54/2022). *Mdr2*-deficient mice were used between 12-14 weeks of age at the start of the experiment. Aged *Mdr2*-deficient mice were 24 weeks at the start of the experiment.

### 2.3 Induction of dextran sodium sulfate colitis

Mice were administered drinking water enriched with 3% DSS for a duration of 7 days, followed by a subsequent 2-day period of pure drinking water without DSS to induce acute DSS colitis (DSS m.w.: 36.000–50.000; MP Biomedicals, Illkirch, France). To induce chronic DSS colitis, mice received drinking water supplemented with 2.5% DSS for three cycles. The first two cycles were five days long, following a remission of 16 days. The third cycle lasted for seven days. The progression of colitis was monitored using endoscopy the next day. The mice were sacrificed and analyzed for pathological conditions of the intestine, as well as the liver one day after endoscopy.

### 2.4 Infection with *Citrobacter rodentium*

Nalidixic acid-resistant, luciferase-expressing derivate of *Citrobacter rodentium* (ICC180) was grown over night in Lysogeny broth (LB) medium, containing 50 µg/ml of nalidixic acid, shaking at 37°C. The suspension of bacteria was washed twice and the concentration was adjusted to 5 x 10^9^ cfu/ml. Mice were infected by oral gavage with 200 µl of *C. rodentium* solution each, containing 1 x 10^9^ cfu. The control group was gavaged with 1x PBS. Seven days after infection, the mice were euthanized, and their intestinal and liver tissues were analyzed to assess pathological conditions.

### 2.5 DDC-induced sclerosing cholangitis

To simulate experimental sclerosing cholangitis through chemical induction, 3,5-diethoxycarbonyl-1,4-dihydrocollidine (DDC; Merk, Germany) was used, by adding 0.1% DDC to the diet. For the preparation of the diet, 1 kg of powdered food was mixed with 1 g DDC and approximately 750 ml water. The food was mixed well, spread onto a board and cut into pieces. The food pellets were dried for 3-4 days and were flipped once a day. Wild type mice aged 10-14 weeks old were fed with the DDC diet for 8 days *ad libitum*. The transaminases were measured as readout for acute inflammation and cell damage in the liver.

### 2.6 Endoscopy

Colonoscopy was performed at specific time intervals to assess the degree of intestinal inflammation as previously described (24), using the Coloview System (Karl Storz, Germany). In summary, anesthetized mice were evaluated endoscopically based on five specific parameters: alterations in the vascularity, thickening of the colon, granularity of the mucosal surface, texture of feces, and visible fibrin, each graded on a scale from 1 to 3, resulting in a cumulative score ranging from 0 (indicating good healthy) and 15 (representing severe colitis).

### 2.7 Fecal transplantation

For murine fecal transplantation, donor mice (DDC-fed mice or DDC+DSS-fed mice) were sacrificed and stool was collected from the colon including the caecum, directly into thioglycolate medium (Merck, Darmstadt, Germany). Samples were homogenized through a 70 μm cell strainer, transferred to brain heart infusion medium (Merck, Darmstadt, Germany), and centrifuged for 10 min at 500g. The supernatant was re-suspended in BHI medium and immediately gavaged into recipient mice. Each mouse was gavaged with 200 µl of stool.

### 2.8 Transaminases

To monitor liver damage, alanine aminotransferase (ALT), aspartate aminotransferase (AST), alkaline phosphatase (ALP), and bilirubin were analyzed in blood serum at the Institute for Experimental Immunology and Hepatology (UKE, Hamburg), via an automated process (COBAS MIRA; Roche, Basel, Switzerland).

### 2.9 Histology

Organs were extracted and preserved in a 4% paraformaldehyde solution for at least 24h. The organs were embedded in paraffin, sectioned, and stained for H&E and Sirius Red. In order to evaluate the liver tissue pathology, both the modified histological activation index (mHAI) and fibrosis were assessed. The mHAI score is composed of five subsets: interface hepatitis (0-4), confluent necrosis (0-6), focal lytic necrosis, apoptosis, focal inflammation (0-4), and portal inflammation (0-4).

### 2.10 Cell isolation

Mice were sacrificed by CO_2_ and O_2_ and immediately perfused with 5mL PBS via the left heart ventricle. Livers were harvested, rinsed in PBS and smashed through a metal strainer. Tissue homogenates were washed with PBS + 1% FBS at 380 × g and 4°C for 10 min. Leukocytes were isolated using a Percoll gradient (GE Healthcare, Uppsala, Sweden). After isolation, cells were stained for flow cytometry.

### 2.11 Flow cytometry

For surface staining, the cells were incubated with the following fluorochrome-conjugated monoclonal antibodies: anti-CD45 (BV510, clone: 30F11) and anti-CD4 (PB, clone: RM4-5) in the presence of a blocking anti-FcgR mAb (clone: 2.4G2) for 20 min at 4°C. Unless otherwise specified, mAbs were purchased from Biolegend (London, England).

For intracellular staining, cell surface markers were stained as described above, followed by fixation of bound antibodies with 4% formalin for 30 min and permeabilization with 0.1% NP-40 for 4 min both at RT. For detection, cells were incubated with PE-conjugated Foxp3 mAb (clone: NRRF-30, eBioscience), AF488-conjugated IL-17A mAb (clone: TC11-18H10.1) and BV785 conjugated INFg mAb (clone: XMG1.2) overnight at 4°C. Cells were analyzed using a Fortessa flow cytometer (BD Biosciences) and FlowJo software (Tree Star, Ashland, OR, USA).

### 2.12 RNA extraction and real-time PCR analysis

Total RNA was extracted from liver tissue using Trizol Reagent (Invitrogen, Waltham MA) and bead beating as previously described (23). For cDNA synthesis, we used the High-Capacity cDNA Reverse Transcription Kit (Thermofisher) with 2mg of RNA per reaction, following the manufacturer’s instructions. For Real-time PCR (RT-PCR) analysis, TaqMan Fast Advanced Master Mix (Thermofisher) was used. The following TaqMan Probes were used: *Col1a1* (Mm00801666_g1), *Acta2* (Mm00725412_s1), *Mmp8* (Mm00439509_m1), *Il6* (Mm00446190_m1), *Il17a* (Mm00439618_m1), *Tnfa* (Mm00443258_m1) and *Hprt* (Mm03024075_m1). Relative expression was normalized to *Hprt*.

### 2.13 Statistics

The human data underwent pre-processing, analysis, and visualization. The analysis was conducted in R using the base statistics and the ggplot2 package (version 3.2.1) for visualization. The Wilcoxon rank sum test/Mann-Whitney *U* test was implemented in the ExactRankTest package (version 0.8). The data are presented as dot plots/violin plots with standard error of the mean +/- standard deviation of the sample mean overlaid as bars. Statistical analysis of the mouse data was conducted using GraphPad Prism software. The Mann-Whitney *U* test was used to determine statistical significance. A p-value <0.05 was used to define significance.

## 3 Results

### 3.1 Induction of DSS colitis attenuates liver pathology in a mouse model of sclerosing cholangitis

**Figure 1:**
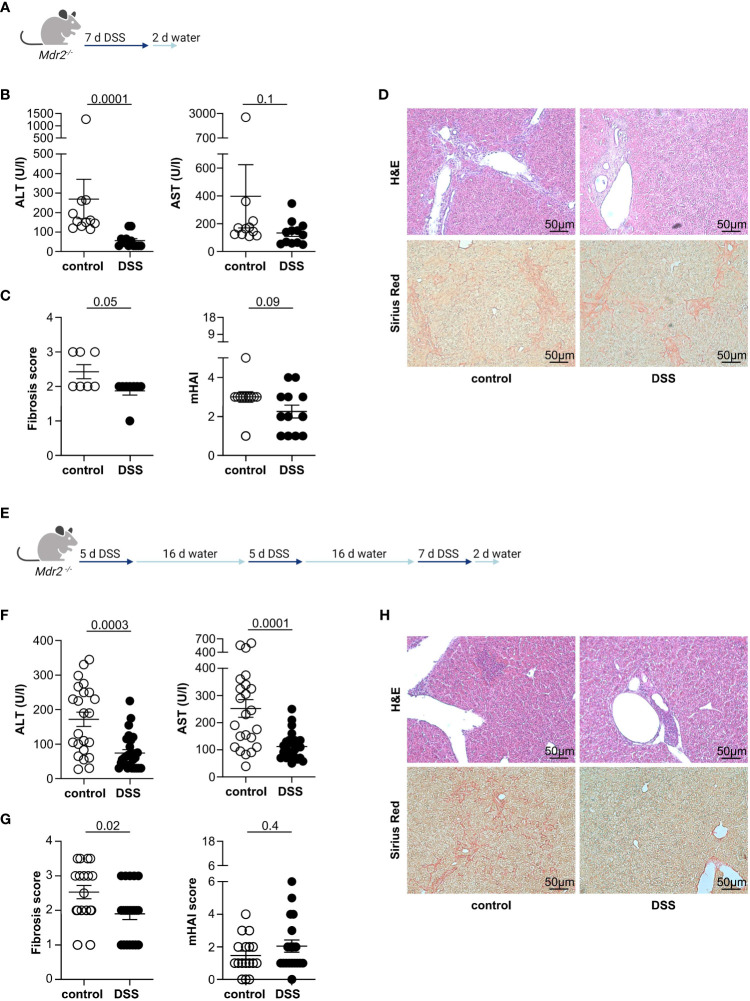
Acute and chronic DSS colitis attenuates liver pathology in *Mdr2*-deficient mice. **(A)** The treatment scheme for acute DSS colitis is shown. **(B)** Liver inflammation after acute DSS colitis was analyzed by serum Alanine Aminotransferase (ALT) and Aspartate Aminotransferase (AST) levels (control n=11, DSS n=12). **(C)** Fibrosis score after acute DSS colitis was analyzed by Sirius Red staining (control n=7, DSS n=8) and mHAI score was analyzed by H&E staining (control n=11, DSS n=12). **(D)** Representative histological liver sections are shown. **(E)** Treatment scheme for chronic DSS colitis. **(F)** Liver inflammation after chronic DSS colitis was analyzed by ALT and AST levels (control n=23, DSS n=27). **(G)** Fibrosis score after chronic DSS colitis was analyzed by Sirius Red staining and mHAI score was analyzed by H&E staining (control n=18, DSS n=21). **(H)** Representative histological liver sections. Mann-Whitney U test was carried out for statistical analysis.

First, we aimed to investigate the connection between sclerosing cholangitis and colitis in experimental mouse models. For this purpose, we induced acute DSS colitis in *Mdr2*-deficient mice, a mouse model for experimental sclerosing cholangitis (25), as depicted in the treatment scheme (**Figure 1A**). Successful colitis development was verified through observed weight loss, endoscopic colitis score, and colon shortening (**Supplementary Figure S1A**). In *Mdr2*-deficient mice with acute DSS colitis, we observed a decrease in serum liver transaminases when compared to *Mdr2*-deficient control animals (**Figure 1B**). Moreover, we also found an attenuation of liver fibrosis in *Mdr2*-deficient mice with acute DSS colitis compared to *Mdr2*-deficient controls. The histological disease activity measured by mHAI score was comparable (**Figures 1C, D**). We isolated lymphocytes from the livers of these mice and found increased frequencies of Foxp3^+^ Treg in *Mdr2*-deficient mice upon acute DSS colitis induction and comparable frequencies of IFNγ^+^ and IL-17A^+^ CD4^+^ T cells (**Supplementary Figure S2A**).

Concomitant IBD in people with PSC presents as chronic, widespread, and mild colitis. To better recapitulate this clinical phenotype, we induced chronic DSS colitis in 12-week-old *Mdr2*-deficient mice through the repeated administration of DSS in the drinking water. The treatment scheme is shown in **Figure 1E**. As expected, the mice developed colitis as shown by weight loss, endoscopic colitis score, and colon shortening (**Supplementary Figure S1B**). Moreover, and consistent with the findings in the acute DSS colitis model, chronic DSS colitis resulted in reduced liver pathology, measured by serum alanine aminotransferase (ALT) and aspartate aminotransferase (AST) levels (**Figure 1F**), and fibrosis as measured by Sirius Red staining in *Mdr2*-deficient mice compared to untreated *Mdr2*-deficient controls. Again, histological disease activity measured by mHAI score was similar (**Figures 1G, H**).

Since sclerosing cholangitis is a slow progressive disease leading to the development of liver cirrhosis or cholangiocarcinoma over time (26), we next aimed to test, whether chronic DSS colitis also attenuates liver inflammation in aged *Mdr2*-deficient mice (**Supplementary Figure S3A**). As expected, aged *Mdr2*-deficient mice developed colitis after repeated administration of DSS through the drinking water as observed by weight loss, endoscopic colitis score, and colon shortening (**Supplementary Figure S3B**). More importantly, aged *Mdr2*-deficient mice showed less pronounced serum ALT levels and AST levels after colitis induction, alongside Bilirubin and alkaline phosphatase (ALP) levels, compared to *Mdr2-*deficient mice without colitis (**Supplementary Figure S3C**). However, the development of fibrosis and the histological disease activity was not significantly attenuated after colitis induction in aged *Mdr2*-deficient mice compared to untreated *Mdr2*-deficient controls (**Supplementary Figures S3D, E**).

Taken together, we found a protective effect of acute and chronic DSS colitis on liver injury in young *Mdr2*-deficient mice. Moreover, in addition to reduced serum transaminases, we showed that the development of liver fibrosis was attenuated. However, this protective effect was less clear in aged *Mdr2*-deficient mice.

### 3.2 Induction of DSS colitis improves liver injury in DDC-induced sclerosing cholangitis

**Figure 2:**
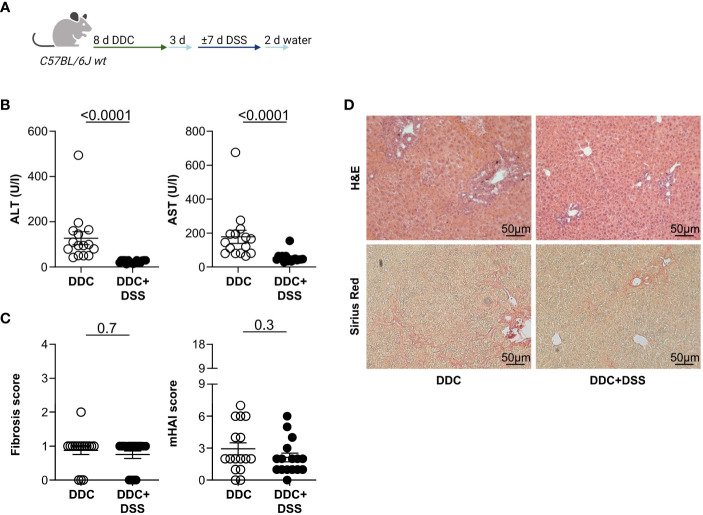
Acute DSS colitis attenuates liver pathology in DDC-induced sclerosing cholangitis. **(A)** Treatment scheme of DDC feeding and acute DSS colitis. **(B)** Liver inflammation after acute DSS colitis was analyzed by serum Alanine Aminotransferase (ALT) and Aspartate Aminotransferase (AST) levels (DDC n=15, DDC+DSS n=16). **(C)** Fibrosis score after acute DSS colitis was analyzed by Sirius Red staining and mHAI score was analyzed by H&E staining (DDC n=16, DDC+DSS n=16). **(D)** Representative histological liver sections. Mann-Whitney U test was carried out for statistical analysis.

Next, we aimed to validate our observation that DSS colitis attenuated liver injury in *Mdr2-*deficient mice in a second model of experimental sclerosing cholangitis. To induce liver pathology, we fed C57BL/6J wild type mice with a 3,5-diethoxycarbonyl-1,4-dihydrocollidine (DDC) diet for eight days, followed by a normal chow diet, *ad libitum*. After three days, acute DSS colitis was induced in these mice, as described above (**Figure 2A**). As expected, the DDC diet alone did not induce colitis. Additional administration of DSS in drinking water induced colitis, as shown by endoscopic scoring methods, weight loss, and colon shortening (**Supplementary Figure S4A**). More importantly, liver inflammation induced by DDC was reduced in mice that had received DDC+DSS, based on serum ALT and AST levels, compared to DDC-fed controls (**Figure 2B**). However, there were no differences in liver fibrosis scores and the histological disease activity detectable between DDC-fed mice and DDC-fed mice with DSS colitis (**Figures 2C, D**). We isolated lymphocytes from the livers of DDC-fed mice with and without acute DSS colitis. We found significantly increased IL-17A^+^ CD4^+^ T cells, after DDC+DSS treatment while frequencies of Foxp3^+^ Treg, and IFNγ^+^ CD4^+^ T cells were comparable between DDC treated mice with and without acute DSS colitis (**Supplementary Figure S2B**).

The liver tissue of *Mdr2*-deficient mice and DDC fed mice upon DSS colitis induction was assessed by RT-PCR for fibrogenesis and inflammatory genes (**Supplementary Figure S5**). Reduced expression of *Col1a1* was detected after colitis induction, which is associated with reduced fibrosis. In addition, increased *Mmp8* expression was found in the groups with colitis. It was reported that overexpression of *Mmp8* is associated with reduced fibrosis (27). No differences were found in the expression of *Acta2* (**Supplementary Figure S5A**). We also found an increase in the expression of the inflammatory genes *Il6* and *Tnfα* in *Mdr2*-deficient mice after DSS colitis, which is in line with our previous study (13) indicating that DSS colitis induces an infiltration of the liver with inflammatory cells (**Supplementary Figure S5B**). These changes were not detectable in the DDC model upon DSS colitis (**Supplementary Figures S5C, D**).

Overall, we found that induction of DSS colitis does not promote cholangitis, but rather attenuates liver injury in different mouse models.

### 3.3 Infection with *Citrobacter rodentium* improves hepatocyte injury in Mdr2-deficient mice

**Figure 3:**
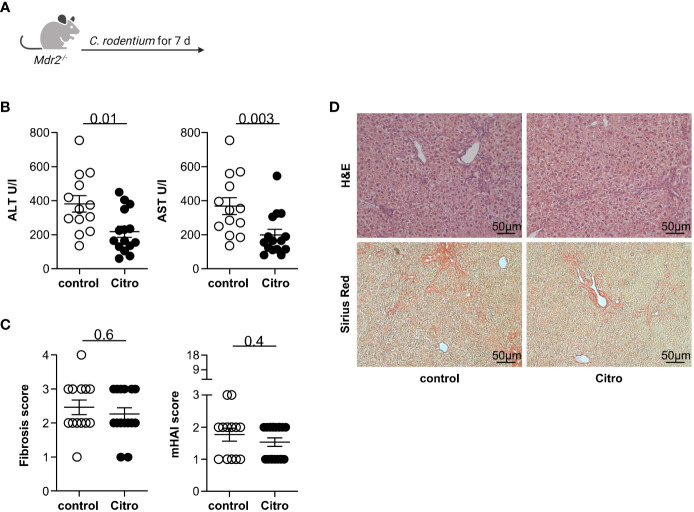
*Citrobacter rodentium* infection reduces hepatocyte injury in Mdr2-deficient mice. **(A)** Treatment scheme of *Citrobacter rodentium* infection. **(B)** Liver inflammation after *C. rodentium* infection was analyzed by serum Alanine Aminotransferase (ALT) and Aspartate Aminotransferase (AST) levels (control n=13, Citro n=15). **(C)** Fibrosis score after *C. rodentium* infection was analyzed by Sirius Red staining and mHAI score was analyzed by H&E staining (DDC n=13, DDC+DSS n=15). **(D)** Representative histological liver sections. Mann-Whitney U test was carried out for statistical analysis.

Next, we aimed to validate our observation that intestinal inflammation attenuates liver injury in mouse models of sclerosing cholangitis using *Citrobacter rodentium* infection as an alternative model for intestinal inflammation (28). Thus, we infected *Mdr2*-deficient mice with *C. rodentium* (**Figure 3A**). Successful infection was assessed by CFU in the caecum content (**Supplementary Figure S6A**). In accordance with the results from the DSS-induced colitis model, the *C. rodentium* infection resulted in a decrease of serum ALT and AST levels in *Mdr2*-deficient mice compared to controls (**Figure 3B**). The fibrosis score and the histological disease activity measured by mHAI were not reduced in the presence of a *C. rodentium* infection compared to the control (**Figures 3C, D**).

In summary, we found that infection with the intestinal pathogen *C. rodentium* attenuates liver inflammation based on serum transaminases but does not impact liver fibrosis.

### 3.4 The beneficial influence of colitis on liver pathology cannot be transmitted through fecal microbiota transplantation

**Figure 4:**
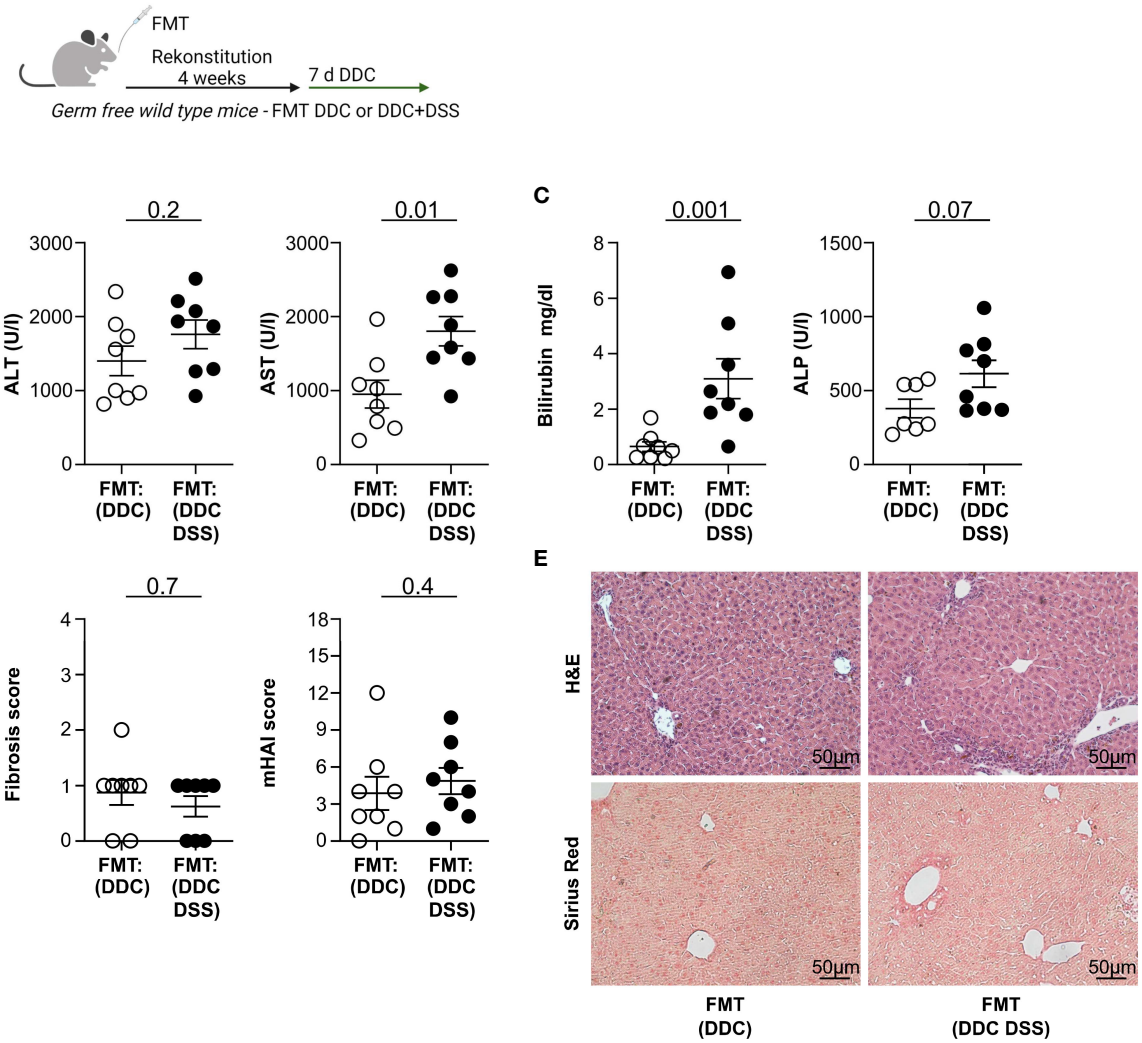
Fecal microbiota transplantation (FMT) of DDC+DSS microbiome in germ-free recipients upon DDC induction results in increased liver pathology compared to DDC microbiome. **(A)** Treatment scheme of the experiment. **(B)** Liver inflammation after DDC feeding was analyzed by serum Alanine Aminotransferase (ALT) and Aspartate Aminotransferase (AST) levels and **(C)** Bilirubin and Alkaline Phosphatase (ALP) levels (FMT : DDC n=8, FMT : DDC DSS n=8). **(D)** Fibrosis score after DDC feeding was analyzed by Sirius Red staining and mHAI score was analyzed by H&E staining (FMT : DDC n=8, FMT : DDC DSS n=8). **(E)** Representative histological liver sections. Mann-Whitney U test was carried out for statistical analysis.

It has recently been suggested that colitis reduces liver injury by modifications of the intestinal microbiota (18). Thus, we next aimed to test whether the protective impact of intestinal inflammation on liver pathology is transmittable through the intestinal microbiota. To this end, we reconstituted germ-free wild type mice with stool derived from mice with DDC-induced sclerosing cholangitis and concomitant DSS colitis, or with DDC-induced sclerosing cholangitis alone. Upon reconstitution, we induced sclerosing cholangitis in these mice by feeding them a DDC diet (see treatment scheme in **Figure 4A**). Gnotobiotic mice, which received microbiota from mice with cholangitis and colitis, developed higher serum ALT, AST, Bilirubin, and ALP levels upon DDC feeding compared to gnotobiotic-mice that had received microbiota from mice with cholangitis alone (**Figures 4B, C**). Fibrosis of the liver was examined by Sirius Red staining and histological disease activity measured by mHAI. No significant distinctions were observed among the groups (**Figures 4D, E**).

Thus, the protective influence of colitis on the severity of liver inflammation is not transmittable via fecal microbiota transplantation.

### 3.5 Intestinal inflammation is linked to a decrease in liver fibrosis among individuals with PSC

**Figure 5:**
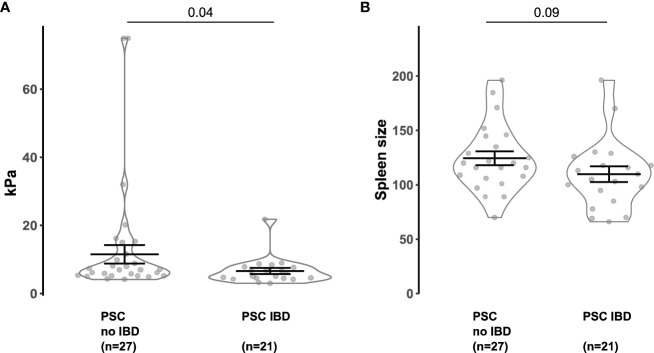
Concomitant IBD attenuates the FibroScan and spleen size in PSC patients. **(A)** FibroScan (Echo-Sense, Paris, France) of PSC patients without IBD and PSC-IBD patients with active IBD. **(B)** Spleen size of PSC patients without IBD and PSC-IBD patients with active IBD. Mann-Whitney U test was carried out for statistical analysis.

Finally, we analyzed a patient cohort to investigate the attenuation of colitis on liver pathology in human individuals. We compared the fibrosis severity and the spleen size (6) of 27 people with PSC alone and 21 people with PSC and active IBD (see **Supplementary Table S1** for the clinical information). We found that liver stiffness values measured by FibroScan (21) were significantly reduced in individuals with PSC-IBD compared to PSC alone (**Figure 5A**). Correspondingly, spleen size was also decreased in individuals with PSC-IBD, compared to PSC without IBD (**Figure 5B**).

Thus, we found reduced liver fibrosis in people with PSC that suffer from concomitant IBD compared to people with PSC alone.

## 4 Discussion

A robust correlation exists between PSC and IBD, as these two diseases frequently occur together. However, it is unclear if IBD would drive PSC, thereby causing this association. Indeed, the current literature is controversial regarding this point. On the one hand, experimental data indicate that barrier defects during colitis cause bacterial translocation, thereby leading to immune cell activation in the liver and immune cell migration to the liver (12–14). These data support the hypothesis that IBD drives PSC. On the other hand, it was recently shown that colitis ameliorates cholestatic liver disease in mice and that the presence of intestinal inflammation is linked to an extended period of liver transplantation-free survival in patients with PSC (18), suggesting that IBD rather protects against PSC.

Intestinal microbiota and bacterial products are known to promote immune responses during both IBD and PSC. Thus, one possible explanation for the observation that IBD ameliorates PSC could be an animal facility specific intestinal microbiota composition that favors a mild course of liver inflammation. To rule out this possibility, we induced DSS colitis in *Mdr2*-deficient mice in our mouse facility. Consistent with the findings of Gui et al., our research demonstrated that induction of acute DSS colitis in *Mdr2*-deficient mice diminished the severity of sclerosing cholangitis. Moreover, induction of a more physiological chronic DSS colitis in *Mdr2*-deficient mice also attenuated liver pathology. Another possible explanation for the reduced liver pathology in the presence of colitis could be the DSS colitis model, since DSS has been detected in the liver upon oral administration (29) and has been suggested to affect the liver (19, 20). To this end, we infected *Mdr2*-deficient mice with *Citrobacter rodentium*. In line with our previous results in DSS colitis, *C. rodentium* infection resulted in reduced serum liver transaminases although liver fibrosis was not improved. The latter could be due to the overall milder intestinal inflammation upon *C. rodentium* infection compared to the DSS model that mainly affects the caecum of these mice and is therefore associated with a lower permeability of the intestine. Also, the genetic model of sclerosing cholangitis using *Mdr2*-deficient mice could impact the PSC severity upon colitis. However, induction of DSS colitis after feeding a DDC diet reduced DDC-mediated liver pathology. Thus, using these different models, we were able to confirm, that the protective effect of IBD on PSC is neither dependent on an animal facility-specific intestinal microbiota nor on the disease model.

However, we found that the protective effect was reduced when the mice were older, and therefore already in the chronic phase of sclerosing cholangitis. One possible explanation would be that the liver fibrosis in old *Mdr2*-deficient mice has already plateaued, and hence, the induction of colitis only has a mild effect. Further studies are warranted to address this point.

The gut-liver axis has been proposed to have a significant role in the progression of sclerosing cholangitis. In the course of intestinal inflammation, the integrity of the gut barrier is compromised, allowing gut microbes, their byproducts, and immune cells to breach the gut barrier and migrate through the portal vein to the liver, where they have the potential to trigger inflammation (12–14, 30–32). These mechanisms may also be involved in the protective influence of colitis against the onset of sclerosing cholangitis. Indeed, it has recently been shown that the protective effect of intestinal inflammation on sclerosing cholangitis may be driven by hepatocellular NF-κB activation induced by lipopolysaccharides, which in turn inhibits bile acid metabolism (18). However, further mechanistic studies are warranted in order to comprehensively investigate the involvement of the gut-liver axis in mediating this protective effect.

The intestinal microbiota is one possible key factor impacting the development of PSC and IBD. We have previously shown a translocation of both immune cells and bacteria from the intestine into the liver during episodes of intestinal inflammation in mouse models (13). However, whether these alterations contribute to, or protect from the development of PSC, remained unclear. For example, the microbiota has a crucial effect on bile acid (BA) synthesis. While it was shown that the microbiota plays a crucial role in restraining BA synthesis through the FXR-FGF15 axis in *Mdr2*-deficient mice, controversial data indicate that individuals with advanced PSC exhibited suppressed BA synthesis, which was linked to an unfavorable prognosis (33). This suggests that the microbiota and the bile acid synthesis may potentially exert a significant influence on the severity of the disease. We used the fecal content from the intestine of mice with only experimental sclerosing cholangitis, or with experimental sclerosing cholangitis and colitis, and transferred it into germ-free recipient wild type mice. We found that the protective effect was not transmitted through fecal microbiota transfer. Surprisingly, we found that the fecal microbiota of mice with sclerosing cholangitis and colitis did indeed rather promote the susceptibility to sclerosing cholangitis compared to the microbiota from mice with sclerosing cholangitis alone. These data indicate that the protective effect of colitis on cholangitis may not be mediated via the intestinal microbiota. However, the reasons for the increased cholangitis susceptibility could not be uncovered in our study. Since Gui et al., 2023 found that bile acids play a major role in the protective effect of colitis on sclerosing cholangitis (18), one could speculate that the altered microbiota impacts the bile acid pool negatively. However, further studies will be essential to test this hypothesis.

In addition, in a recent investigation, fecal microbiota samples from people with PSC and UC were transferred into germ-free mice. Indeed, germ-free mice receiving fecal microbiota samples of people with PSC and UC developed increased liver pathology upon induction of DDC treatment compared to mice receiving fecal microbiota from healthy controls (14). People with PSC exhibit a distinct microbiota composition in comparison to healthy controls and individuals with PSC-IBD (6). Thus, further studies comparing these groups would also be important.

It has been described that recurrent PSC after liver transplantation is more frequent in individuals with PSC-IBD in contrast to those with PSC without intestinal inflammation (34, 35). There are publications showing that this increased recurrence rate is reduced upon colectomy (36, 37). The reasons for this are unclear and may be due to the immunosuppressive therapy. Even if these data are controversial and there are also studies not confirming this, we speculate, based on our data, that the gut microbiota could potentially play a role in this phenomenon, as we found that the microbiota of colitogenic mice rather drives sclerosing cholangitis. However, further translational studies are needed to test these hypotheses.

Finally, analyzing a recently published cohort of PSC patients (6), we found that people with active PSC-IBD individuals show decreased levels of liver fibrosis compared to people with PSC without associated IBD. The liver fibrosis in people with PSC-IBD was assessed by the measurement of the liver stiffness using fibroscan, since human liver biopsies are not taken routinely in these patients. This method has been shown to be a reliable non-invasive tool to assess liver fibrosis in various chronic liver diseases (21).

These data are also in line with the data from Gui et al., indicating that intestinal inflammation improves liver transplantation-free survival in people with PSC (18).

Overall, our study provides insights into the relationship between PSC and IBD. Our experimental mouse data indicate that colitis does not, per se, promote sclerosing cholangitis, but may rather protect against it. In line with this, people with PSC and concomitant active IBD show less severe liver fibrosis compared to people with PSC without IBD.

## Data availability statement

The original contributions presented in the study are included in the article/**Supplementary Material**, further inquiries can be directed to the corresponding authors.

## Ethics statement

The studies involving humans were approved by ethical commission of the medical association Hamburg. The studies were conducted in accordance with the local legislation and institutional requirements. The participants provided their written informed consent to participate in this study. The animal study was approved by Behörde für Justiz und Verbraucherschutz, Hamburg, Germany. The study was conducted in accordance with the local legislation and institutional requirements.

## Author contributions

FS: Conceptualization, Data curation, Formal Analysis, Investigation, Writing – original draft. NS: Conceptualization, Data curation, Formal Analysis, Investigation, Writing – review & editing. BS: Data curation, Formal Analysis, Writing – review & editing. FM: Data curation, Formal Analysis, Writing – review & editing. MN: Writing – review & editing. MS: Writing – review & editing. SS-W: Writing – review & editing. EG: Writing – review & editing. JK: Writing – review & editing. TF: Resources, Writing – review & editing. SW: Resources, Writing – review & editing. CS: Writing – review & editing. NG: Writing – review & editing. SH: Conceptualization, Funding acquisition, Project administration, Supervision, Writing – original draft. TB: Conceptualization, Project administration, Supervision, Writing – original draft.

## Supplementary material

Supplementary Figure S1 Acute and chronic DSS colitis induction in *Mdr2*-deficient mice. **(A)** Weight loss, endoscopic colitis score, and colon length after acute DSS colitis in *Mdr2*-deficient mice (control n=11, DSS n=12). **(B)** Weight loss, endoscopic colitis score, and colon length after chronic DSS colitis in *Mdr2*-deficient mice (control n=23, DSS n=27).

Supplementary Figure S2 Frequency of T helper cell subsets in the liver of *Mdr2*-deficient mice or DDC mice upon acute DSS colitis. **(A)** Shown are the frequency of whole CD4^+^, IFNg^+^, IL-17A^+^ and Foxp3^+^ T cells in the liver of *Mdr2*-deficient mice after DSS colitis. **(B)** Shown are the frequency of whole CD4^+^, IFNg^+^, IL-17A^+^ and Foxp3^+^ T cells in the liver of DDC treated mice after DSS colitis. For statistical analysis26, Mann-Whitney U test was performed (p<0.05). Lines indicate mean ± SEM.

Supplementary Figure S3 Aged *Mdr2*-deficient mice are no longer protected after chronic DSS colitis. **(A)** Weight loss, endoscopic colitis score, and colon length after chronic DSS colitis in aged *Mdr2*-deficient mice (control n=8, DSS n=8). **(B)** Liver inflammation after chronic DSS colitis was analyzed by ALT, AST, Bilirubin, and ALP levels (control n=8, DSS n=8). **(C)** Fibrosis score after chronic DSS colitis was analyzed by Sirius Red staining and mHAI score was analyzed by H&E staining (control n=8, DSS n=8). **(D)** Representative histological liver sections.

Supplementary Figure S4 Acute DSS colitis induction in DDC-fed mice. **(A)** Weight loss, endoscopic colitis score, and colon length after acute DSS colitis in DDC-fed mice (control n=16, DSS n=16). **(B)** Weight loss, endoscopic colitis score, and colon length after chronic DSS colitis in *Mdr2*-deficient mice (control n=16, DSS n=16).

Supplementary Figure S5 Expression of fibrosis and inflammatory associated genes in the liver. (A-B) Shown is the gene expression in the liver of *Mdr2*-deficient mice after acute DSS colitis induction. (C-D) Shown is the gene expression in the liver of DDC fed wild type mice after acute DSS colitis induction. The gene expression was normalized to the housekeeping gene Hprt. For statistical analysis, Mann-Whitney U test was performed (p≤0.05). Lines indicate mean ± SEM.

Supplementary Figure S6 Infection of *Mdr2*-deficient mice with C.rodentium. **(A)** CFU of whole caecum content of *Mdr2*-deficient mice 7 days after infection (control n=5, DSS n=5).
