# Supplementary material for: A protective effect of inflammatory bowel disease on the severity of sclerosing cholangitis
Source: Front Immunol. 2024 Mar 6;15:1307297. doi: 10.3389/fimmu.2024.1307297 (PMC10950911; doi:10.3389/fimmu.2024.1307297)
Supplement: Supplementary file 1 [file Table_1.docx]

**Supplementary Table 1**

|  | **PSC IBD IBD score >0** | **PSC no IBD** | **Overall** |
| --- | --- | --- | --- |
| n | 21 | 27 | 48 |
| UC/CD/undefined (%) | 16/3/2 (76%/14%/10%) |  |  |
| age in years (median, range) | 33 [19-69] | 46 [20-74] | 40 [19-74] |
| years since PSC diagnosis (median, range) | 9 [0-33] | 6 [0-17] | 8 [0-33] |
| smoking (% of known) | 0 (0%) | 4 (27%) | 4 (18%) |
| sex male (%) | 16 (76%) | 19 (70%) | 35 (73%) |
|  |  |  |  |
| **Medications, n (%)** |  |  |  |
| 5-ASA | 9 (43%) | 1 (4%) | 10 (21%) |
| Thiopurines | 5 (24%) | 4 (15%) | 9 (19%) |
| Anti-TNF | 11 (52%) | 0 (0%) | 11 (23%) |
| UDCA | 16 (76%) | 22 (81%) | 38 (79%) |
| Steroids | 1 (5%) | 7 (26%) | 8 (17%) |
