# Supplementary figures and images for: A protective effect of inflammatory bowel disease on the severity of sclerosing cholangitis
Source: Front Immunol. 2024 Mar 6;15:1307297. doi: 10.3389/fimmu.2024.1307297 (PMC10950911; doi:10.3389/fimmu.2024.1307297)

**A**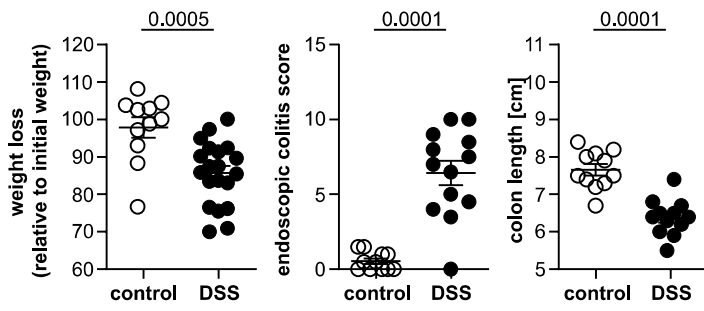**B**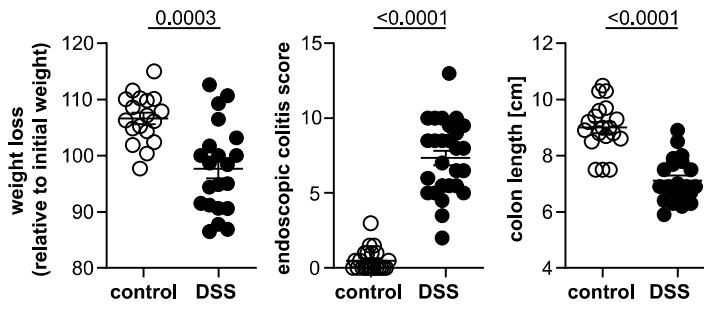

Supplement: Supplementary Figure S1 — Acute and chronic DSS colitis induction in Mdr2-deficient mice. (A) Weight loss, endoscopic colitis score, and colon length after acute DSS colitis in Mdr2-deficient mice (control n=11, DSS n=12). (B) Weight loss, endoscopic colitis score, and colon length after chronic DSS colitis in Mdr2-deficient mice (control n=23, DSS n=27). [file Image_1.pdf]

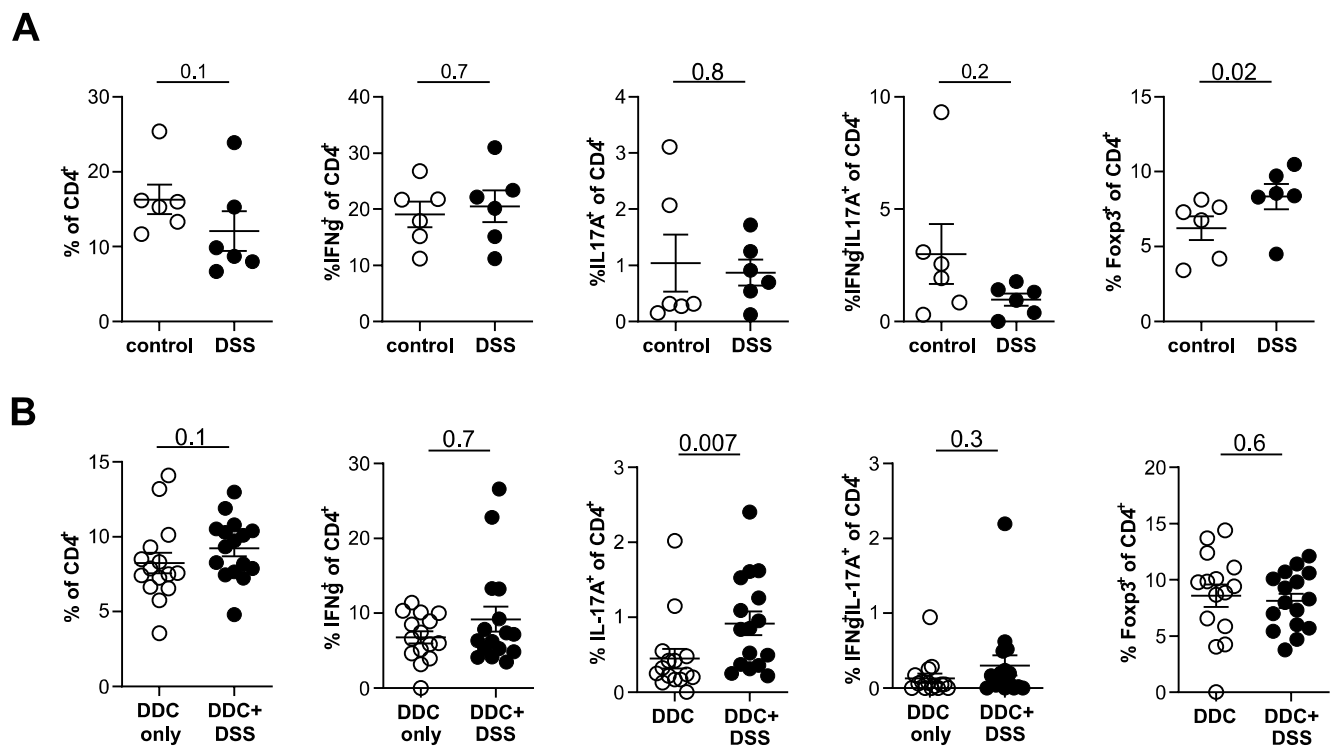

Supp. Figure 2

Supplement: Supplementary Figure S2 — Frequency of T helper cell subsets in the liver of Mdr2-deficient mice or DDC mice upon acute DSS colitis. (A) Shown are the frequency of whole CD4+, IFNg+, IL-17A+ and Foxp3+ T cells in the liver of Mdr2-deficient mice after DSS colitis. (B) Shown are the frequency of whole CD4+, IFNg+, IL-17A+ and Foxp3+ T cells in the liver of DDC treated mice after DSS colitis. For statistical analysis26, Mann-Whitney U test was performed (p<0.05). Lines indicate mean ± SEM. [file Image_2.pdf]

**A**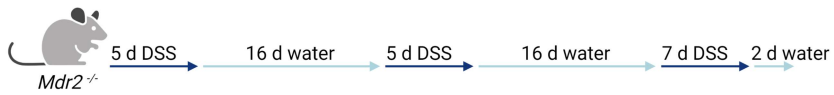**B**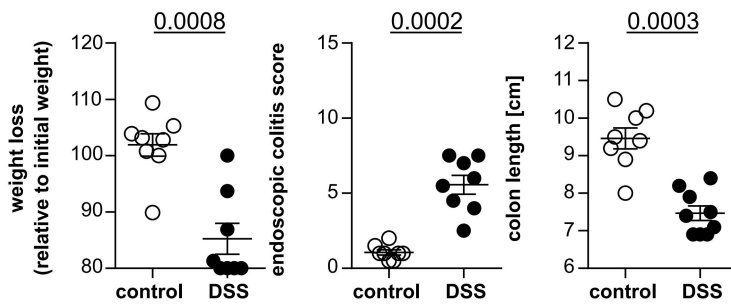**C**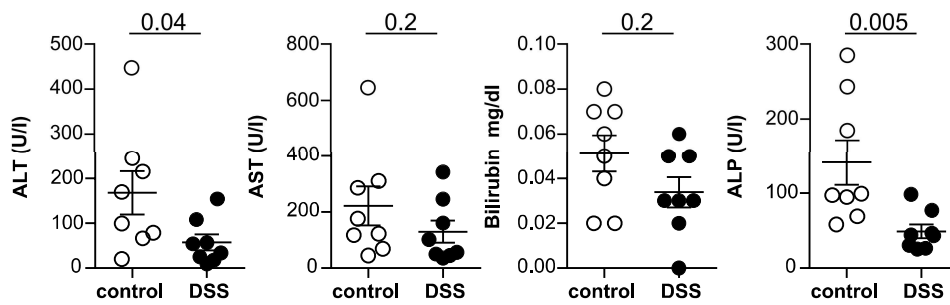**D**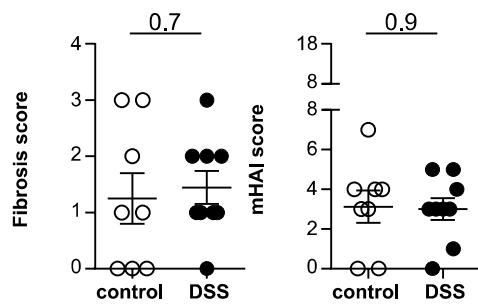**E**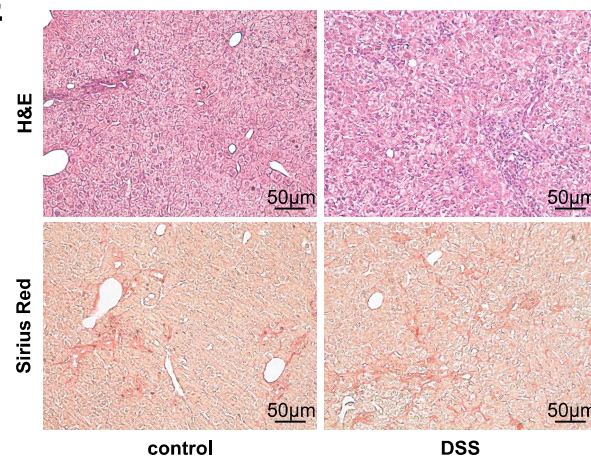

Supplement: Supplementary Figure S3 — Aged Mdr2-deficient mice are no longer protected after chronic DSS colitis. (A) Weight loss, endoscopic colitis score, and colon length after chronic DSS colitis in aged Mdr2-deficient mice (control n=8, DSS n=8). (B) Liver inflammation after chronic DSS colitis was analyzed by ALT, AST, Bilirubin, and ALP levels (control n=8, DSS n=8). (C) Fibrosis score after chronic DSS colitis was analyzed by Sirius Red staining and mHAI score was analyzed by H&E staining (control n=8, DSS n=8). (D) Representative histological liver sections. [file Image_3.pdf]

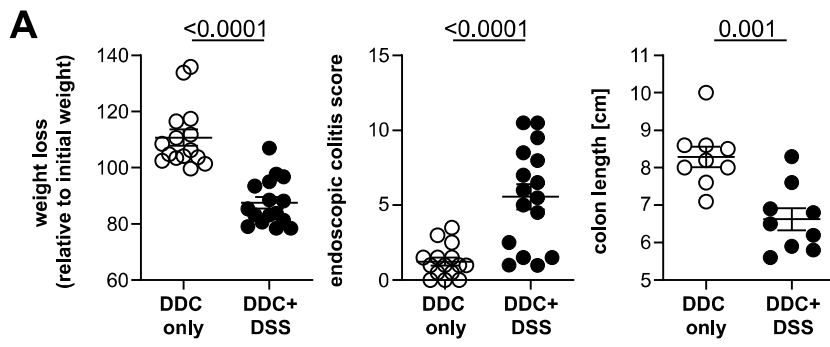

Supp. Figure 4

Supplement: Supplementary Figure S4 — Acute DSS colitis induction in DDC-fed mice. (A) Weight loss, endoscopic colitis score, and colon length after acute DSS colitis in DDC-fed mice (control n=16, DSS n=16). (B) Weight loss, endoscopic colitis score, and colon length after chronic DSS colitis in Mdr2-deficient mice (control n=16, DSS n=16). [file Image_4.pdf]

**A**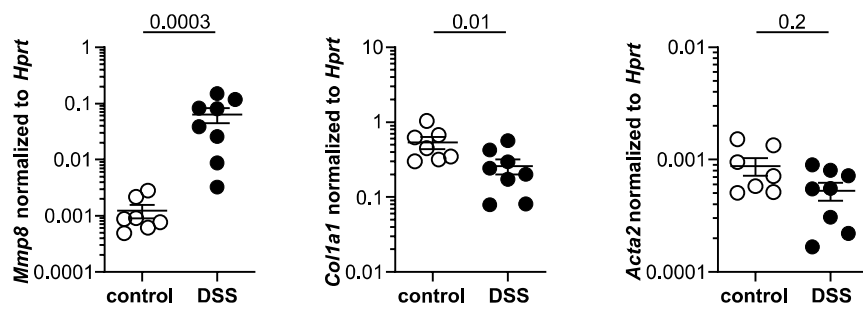**B**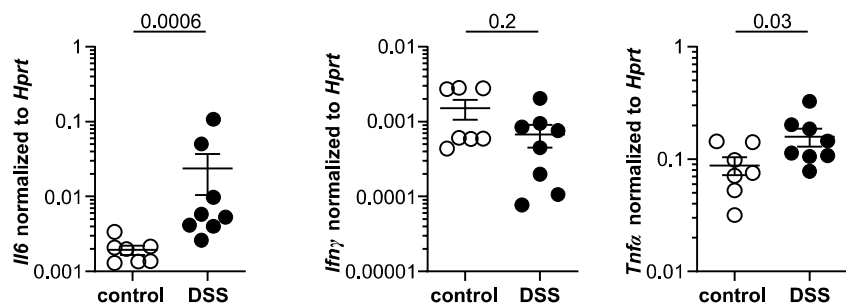**C**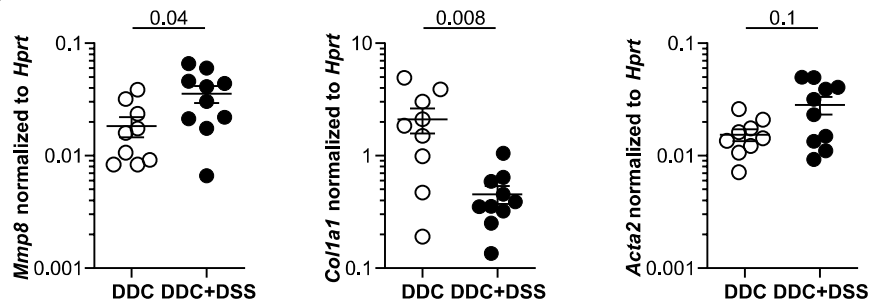**D**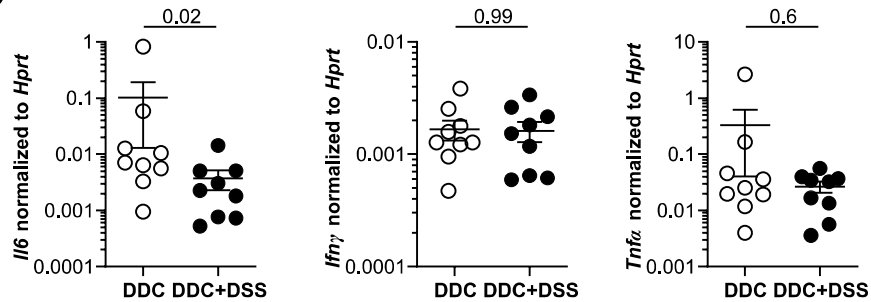

Supp. Figure 5

Supplement: Supplementary Figure S5 — Expression of fibrosis and inflammatory associated genes in the liver. (A-B) Shown is the gene expression in the liver of Mdr2-deficient mice after acute DSS colitis induction. (C-D) Shown is the gene expression in the liver of DDC fed wild type mice after acute DSS colitis induction. The gene expression was normalized to the housekeeping gene Hprt. For statistical analysis, Mann-Whitney U test was performed (p≤0.05). Lines indicate mean ± SEM. [file Image_5.pdf]

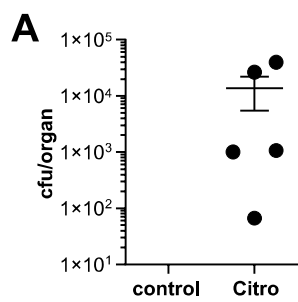

Supp. Figure 6

Supplement: Supplementary Figure S6 — Infection of Mdr2-deficient mice with C.rodentium. (A) CFU of whole caecum content of Mdr2-deficient mice 7 days after infection (control n=5, DSS n=5). [file Image_6.pdf]
